# LPC-SonoNet: A Lightweight Network Based on SonoNet and Light Pyramid Convolution for Fetal Ultrasound Standard Plane Detection

**DOI:** 10.3390/s24237510

**Published:** 2024-11-25

**Authors:** Tianxiang Yu, Po-Hsiang Tsui, Denis Leonov, Shuicai Wu, Guangyu Bin, Zhuhuang Zhou

**Affiliations:** 1 Department of Biomedical Engineering, College of Chemistry and Life Science, Beijing University of Technology, Beijing 100124, China; ytx_pedestrian@163.com (T.Y.); wushuicai@bjut.edu.cn (S.W.); 2 Department of Medical Imaging and Radiological Sciences, College of Medicine, Chang Gung University, Taoyuan 333323, Taiwan; tsuiph@mail.cgu.edu.tw; 3 Division of Pediatric Gastroenterology, Department of Pediatrics, Chang Gung Memorial Hospital at Linkou, Taoyuan 333423, Taiwan; 4 Liver Research Center, Chang Gung Memorial Hospital at Linkou, Taoyuan 333423, Taiwan; 5 Research Center for Radiation Medicine, Chang Gung University, Taoyuan 333323, Taiwan; 6 Research and Education Laboratory, Research and Practical Clinical Center for Diagnostics and Telemedicine Technologies of the Moscow Health Care Department, Moscow 127051, Russia; strat89@mail.ru; 7 Department of Fundamentals of Radio Engineering, Moscow Power Engineering Institute, Moscow 111250, Russia; 8 Department 41, Federal Research Center “Computer Science and Control” of the Russian Academy of Sciences, Moscow 119333, Russia

**Keywords:** fetal ultrasound, standard plane, deep learning, image classification, convolutional neural network

## Abstract

The detection of fetal ultrasound standard planes (FUSPs) is important for the diagnosis of fetal malformation and the prevention of perinatal death. As a promising deep-learning technique in FUSP detection, SonoNet’s network parameters have a large size. In this paper, we introduced a light pyramid convolution (LPC) block into SonoNet and proposed LPC-SonoNet with reduced network parameters for FUSP detection. The LPC block used pyramid convolution architecture inspired by SimSPPF from YOLOv6 and was able to extract features from various scales with a small parameter size. Using SonoNet64 as the backbone, the proposed network removed one of the convolutional blocks in SonoNet64 and replaced the others with LPC blocks. The proposed LPC-SonoNet model was trained and tested on a publicly available dataset with 12,400 ultrasound images. The dataset with six categories was further divided into nine categories. The images were randomly divided into a training set, a validation set, and a test set in a ratio of 8:1:1. Data augmentation was conducted on the training set to address the data imbalance issue. In the classification of six categories and nine categories, LPC-SonoNet obtained the accuracy of 97.0% and 91.9% on the test set, respectively, slightly higher than the accuracy of 96.60% and 91.70% by SonoNet64. Compared with SonoNet64 with 14.9 million parameters, LPC-SonoNet had a much smaller parameter size (4.3 million). This study pioneered the deep-learning classification of nine categories of FUSPs. The proposed LPC-SonoNet may be used as a lightweight network for FUSP detection.

## 1. Introduction

Fetal malformation is the structural and functional abnormality of the body due to factors including but not limited to the environment, infection, and diabetes [1]. Fetal malformation can lead to perinatal death and life-long disability [2]. Hence, prenatal diagnosis and management are necessary. The methods of prenatal diagnosis mainly include magnetic resonance imaging (MRI) and ultrasound screening techniques [3,4]. The ultrasound screening is widely used due to its advantages of a low cost and real-time visualization capability [5,6]. During the ultrasound imaging, the biometric parameters such as the biparietal diameter and head circumference can be used to detect fetal development and diagnose fetal malformation [7]. Therefore, the fetal ultrasound standard planes for measuring such biometric parameters ought to be paid much attention for the reproducibility of the diagnosis [7,8]. In clinical settings, the way to obtain standard planes mainly depends on clinicians’ manual selection while scanning the pregnant women with ultrasound probes [9]. In addition, the variety of standard planes and interference anatomical structures similar to those in the standard planes brings challenges to manual detection [6,8].

In recent years, deep-learning techniques including convolutional neural networks (CNNs) have been applied to the field of medical image processing including fetal ultrasound standard plane detection. For instance, Chen et al. proposed the multi-organ foundation (MOFO) model for ultrasound images segmentation [10]. Chernyshov et al. introduced a U-Net based network for the segmentation and quantification of echocardiography [11]. Su et al. proposed JANet for the segmentation of the left ventricle in ultrasound videos based on ResNet and U-Net [12]. The standard plane detection has been formulated as the classification of several kinds of standard planes. Chen et al. combined different kinds of networks and proposed N-CNN and T-RNN [13,14]. Baumgartner et al. proposed SonoNet based on the Visual Geometry Group (VGG) network, the accuracy of which achieved 90.1% in the classification of 13 standard planes [15]. Ye et al. proposed a network combining YOLOV3 and ResNeXt [16]. Pu et al. proposed the FUSPR network and achieved an accuracy of 87.38% in the classification of four categories, including the fetal abdominal standard plane, fetal thalamus standard plane, fetal cerebellum standard plane, and fetal lumbosacral spine standard plane [17]. Kong et al. proposed the MSDNet based on DenseNet, which was able to extract features from various scales, and achieved an accuracy of 98.26% [18]. In addition to fetal plane detection, deep learning has been used in other tasks. Lin et al. proposed a method based on Faster R-CNN and MFR-CNN for standard plane and inner tissue detection [19,20]. The USPD proposed by Zhao et al. was able to detect standard planes and simultaneously explain the detection results [8]. Cai et al. proposed the multi-task SonoEyeNet as an AI-powered tool that uses sonographer eye movements to create visual cues that help automate the process of finding the correct abdominal circumference measurement plane in ultrasound exams [21].

In 2020, a dataset [22] was made public by Burgos-Artizzu et al., encouraging the related research in fetal ultrasound standard plane detection. This dataset consists of six categories of standard planes including the fetal abdomen, fetal brain, fetal femur, fetal thorax, maternal cervix, and other. The category of fetal brain can further be divided into four categories: trans-ventricular, trans-thalamic, trans-cerebellum, and other brain standard planes; then, the total number of categories is nine.

With Burgos-Artizzu et al.’s dataset [22], Krishna and Kokil proposed three kinds of deep-learning networks which combined AlexNet, VGG and ResNet, achieving an accuracy of 95.1%, 95.5%, and 95.7%, respectively, in the classification of six categories of standard planes [5,23,24]. In addition to the classification of six categories, some researchers paid attention to the classification of three categories of brain standard planes, i.e., trans-ventricular, trans-thalamic, and trans-cerebellum. Coronado-Gutiérrez et al. used ResNet-18 pretrained by the ImageNet dataset to classify the three categories of brain standard planes, with an accuracy of 98.1% [25]. Vetriselvi and Thenmozhi designed a binary-channel CNN and achieved an accuracy of 97.0% in the same classification task [26]. In addition, some researchers chose to design a model for these two classification tasks (i.e., the classification of six categories of standard planes and the classification of three categories of brain standard planes) at the same time. Annamalai and Sindhu proposed an ensemble network with InceptionResNetV2, DenseNet121, and Xception and achieved an accuracy of 96.9% and 93.7%, respectively, in the classification of six categories and three categories [27]. Zamojski et al. combined EfficientV2 and a recurrent neural network (RNN) to classify three and six categories of standard planes [28].

It can be seen that the ensemble frameworks were preferred in the classification of six categories and achieved excellent performance due to its ability to extract features from various scales. However, the ensemble framework leads to large parameter sizes and a long inference time. In this paper, we proposed a lightweight network based on SonoNet [15] and introduced light pyramid convolution (LPC) blocks inspired by the Simplified Spatial Pyramid Pooling Fast (SimSPPF) from the YOLOv6 [29]. The proposed network was termed LPC-SonoNet, which was trained and tested using Burgos-Artizzu’s dataset [22]. While the Burgos-Artizzu dataset [22] encompasses nine distinct image categories, most of the research has focused on classifying either six or three categories. This preference for a smaller number of categories necessitates additional classification steps to identify specific standard planes, such as the trans-ventricular plane. Recognizing this limitation, we applied the proposed LPC-SonoNet to the classification of all nine categories of fetal ultrasound standard planes. The main contributions of this paper are as follows:

1. We proposed a lightweight deep-learning model based on LPC and SonoNet. Compared to SonoNet, the proposed LPC-SonoNet demonstrates a slight improvement in classifying six categories on the Burgos-Artizzu dataset [22], while simultaneously reducing network complexity (i.e., requiring fewer parameters).
2. The proposed LPC-SonoNet was applied to the classification of nine categories on Burgos-Artizzu’s dataset [22], enabling the direct identification of each of the nine kinds of standard planes.

## 2. Materials and Methods

### 2.1. Dataset

**Figure 1:**
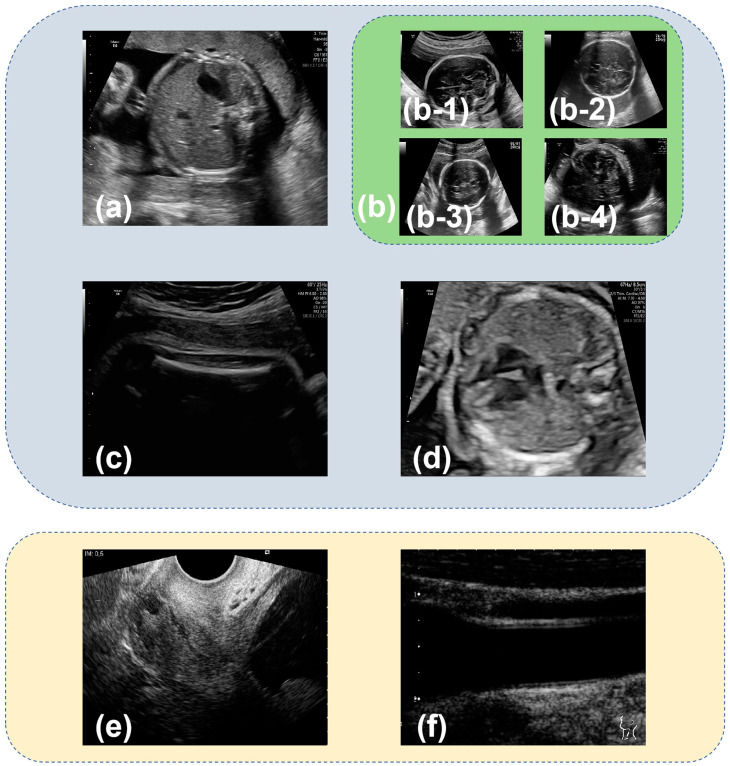
Representative images from Burgos-Artizzu’s dataset [22] depicting various fetal ultrasound standard planes: (**a**) fetal abdominal standard plane; (**b**) fetal brain standard plane; (**b-1**) fetal trans-ventricular standard plane; (**b-2**) fetal trans-thalamic standard plane; (**b-3**) fetal trans-cerebellum standard plane; (**b-4**) other brain standard plane; (**c**) fetal femur standard plane; (**d**) fetal thorax standard plane; (**e**) maternal cervix; and (**f**) other. (**a**–**f**) are samples for six categories classification. (**a**–**f**) are samples for nine categories classification.

**Figure 2:**
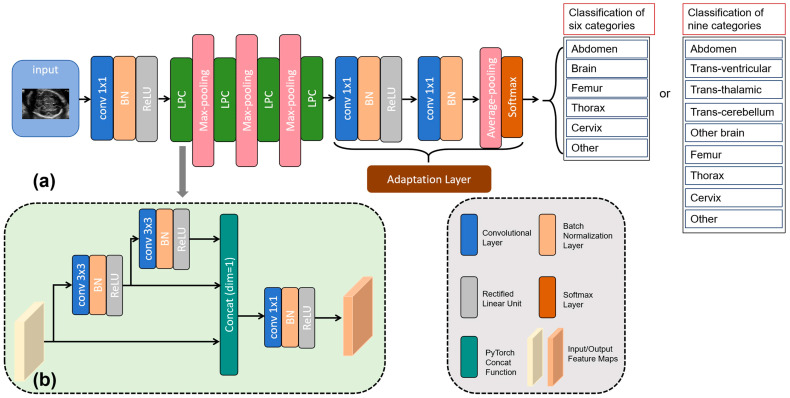
The architecture of the proposed LPC-SonoNet: (**a**) the backbone of LPC-SonoNet; and (**b**) the architecture of the light pyramid convolution (LPC) block. BN: batch normalization; conv: convolution; ReLU: rectified linear unit.

**Figure 3:**
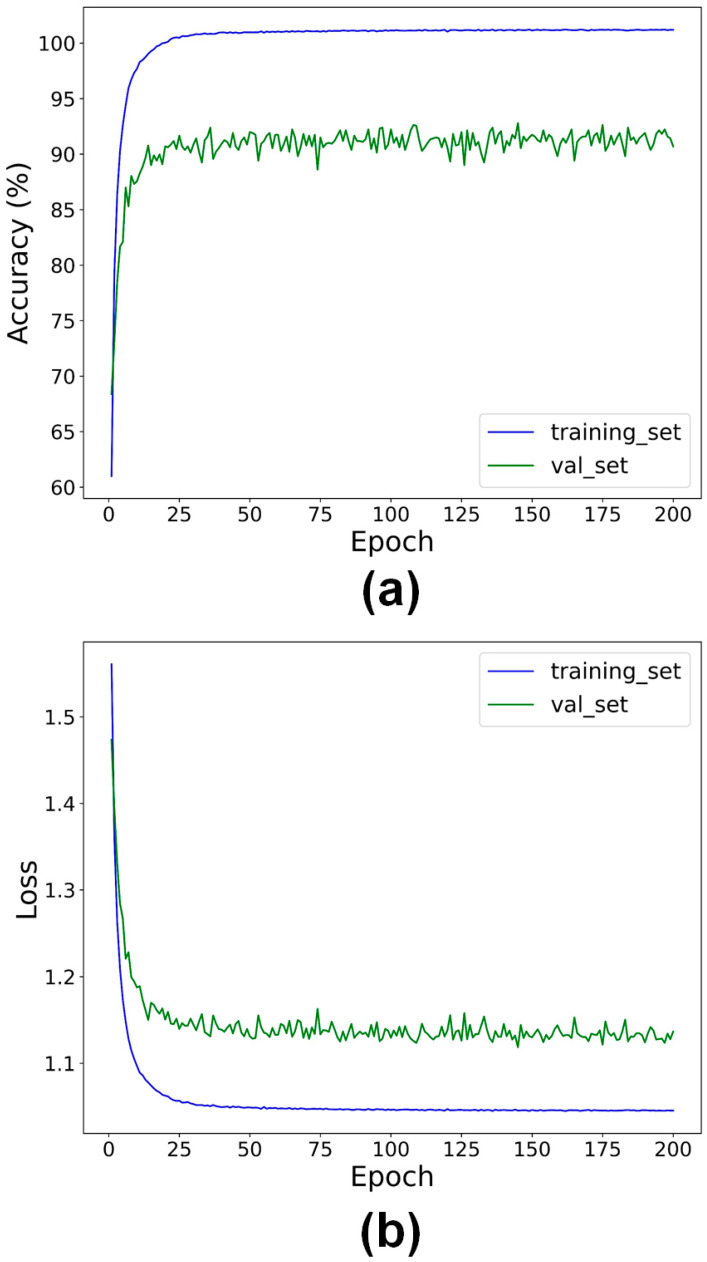
The accuracy (**a**) and loss (**b**) on the training set and validation set as a function of epochs when training the proposed LPC-SonoNet model.

**Figure 4:**
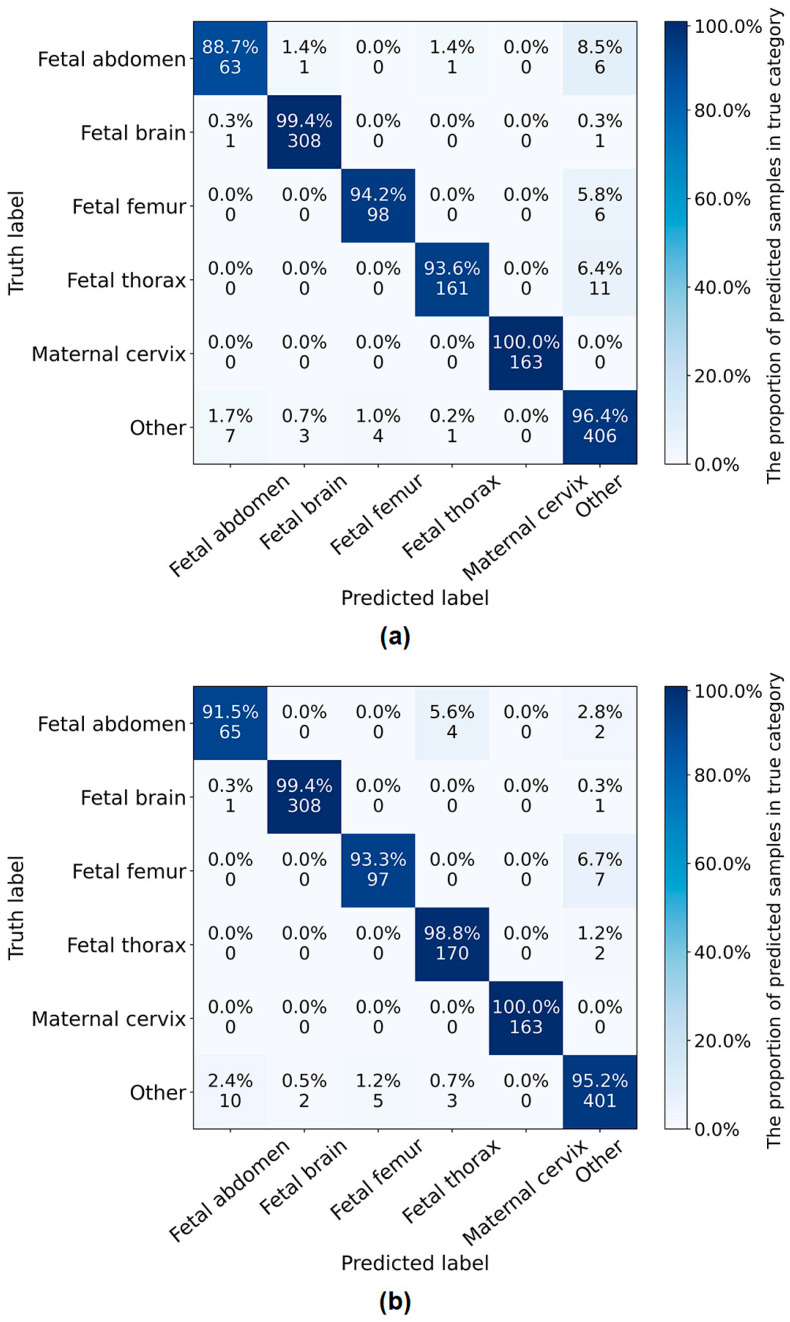
The confusion matrix of six-category classification of standard planes by the proposed network (**b**) and SonoNet64 (**a**).

The open-access dataset used in this paper was made public in 2020 by Burgos-Artizzu et al. [22]. The 12,400 images in the dataset were manually labeled by an expert maternal fetal clinician and divided into 6 categories: fetal abdominal standard plane (Figure 1a), fetal brain standard plane (Figure 1b), fetal femur standard plane (Figure 1c), fetal thorax standard plane (Figure 1d), maternal cervix (Figure 1e), and other (Figure 1f). The fetal brain standard plane was further categorized into fetal trans-ventricular standard plane (Figure 1(b-1)), fetal trans-thalamic standard plane (Figure 1(b-2)), fetal trans-cerebellum standard plane (Figure 1(b-3)), and other brain standard plane (Figure 1(b-4)). The dataset was randomly divided into training, validation, and testing sets in a ratio of 8:1:1, resulting in 9916 images for training, 1243 for validation, and 1241 for testing. The specific number of images used for classifying six and nine categories is outlined in Table 1 and Table 2.

### 2.2. Data Augmentation

The images in the dataset exhibited an imbalance in the number of samples across different categories (Table 1 and Table 2), with the femur and abdominal standard planes having significantly fewer instances than others. To address the data imbalance issue, data augmentation methods including image flipping and rotation, contrast and brightness enhancement, and Gaussian blur were applied only to the training set. The augmented training set for the classification of six and nine categories are shown in Table 3 and Table 4, respectively. In the classification of six categories, each category of image data in the training set was augmented to about 2500 images except for the “other” category. In the classification of nine categories, each category of data in the training set was augmented to around 1300 images except for the “other” category.

### 2.3. Network Architecture

SonoNet [15] is a kind of VGG [30]-based network which has various versions including SonoNet16, SonoNet32, and SonoNet64. Due to its excellent performance in the classification of standard planes and relatively small parameter sizes, SonoNet64 [15], which performed best compared to the other versions of SonoNet, was used as the backbone of the proposed LPC-SonoNet (Figure 2a). In order to obtain a lightweight framework, the LPC block (Figure 2b) inspired by the SimSPPF block from the YOLOv6 [29] was introduced for further reduction of parameter sizes. The proposed LPC-SonoNet consisted of four LPC blocks used for extracting features and one adaptation layer for classification based on feature maps. Compared with SonoNet64 [15], LPC-SonoNet had one fewer block for feature extraction, obtaining smaller parameter sizes.

Compared with its prototype, i.e., the SimSPPF [29], LPC was designed for convolution rather than pooling but they shared the similar architecture. Before the concatenation layer, two convolutional layers with the kernel size of 3 × 3 provided feature maps in large receptive field. The feature maps in the small receptive field and those in the large receptive field were obtained, respectively, by the two 3 × 3 convolutional layers (Figure 2b). These two feature maps, together with the original feature maps, provided the feature maps of three different scales. The concatenation layer was designed for combining feature maps from various scales. A convolutional layer with the kernel size of 1 × 1 was set after the concatenation layer for feature fusion. On the whole, the pyramid architecture enabled the LPC block to extract feature from three receptive fields with three convolutional layers with small kernel sizes.

The adaptation layer (Figure 2a) in the proposed LPC-SonoNet was a block from SonoNet [15]. This layer had two convolutional layers, providing a feature map with K channels, where K was the number of categories. The adaptation layer was designed for taking images with variant sizes as the input and for explicitly searching for the region of interest in the image with the max-pooling layer [15,31].

### 2.4. Experimental Setup

Two groups of experiments were conducted in this study. The first group of experiments was conducted for comparing the performance of LPC-SonoNet and SonoNet64 in the classification of six categories of standard planes. The second was performed for evaluating the performance of the proposed LPC-SonoNet in the classification of nine categories. In these experiments, the LPC-SonoNet models were trained with the augmented training set and tested with the test set.

In the training process, the model was trained with 200 epochs. The initial learning rate was 0.001 and decayed by 10 times each 50 epochs. The optimizer was Adam optimizer [32]. The loss function was the cross entropy. The experiments were conducted on a graphics workstation with Intel(R) Xeon(R) Gold 6132 CPU@2.60 GHz 2.59 GHz (2 processors), NVIDIA TITAN RTX 24 G, and 128 G RAM. PyTorch (version 1.8.1) and Python (version 3.8) were used for the deep-learning framework.

### 2.5. Standard Plane Detection Performance Evaluation Metrics

The metrics used for standard plane detection performance evaluation were the accuracy, sensitivity (i.e., recall), and specificity:

(1) $$\mathrm{Accuracy}=\frac{TP+TN}{TP+TN+FP+FN}$$

(2) $$\mathrm{Sensitivity}=\frac{TP}{TP+FN}$$

(3) $$\mathrm{Specificity}=\frac{TN}{TN+FP}$$

where *TP*, *TN*, *FP*, and *FN* are the true positives, true negatives, false positives, and false negatives, respectively.

## 3. Results

### 3.1. Results of Six-Category Classification

Figure 3 shows the accuracy and loss on the training set and validation set as a function of training epochs. As the epoch increased, both the accuracy of the training and validation sets increased, and the loss of the two sets decreased, illustrating that there was nearly no overfitting when training the LPC-SonoNet model. The small local fluctuations for the accuracy and loss curves of the validation set may be due to the small sizes of the validation set.

Table 5 shows the results of the classification on six categories by the proposed LPC-SonoNet and SonoNet64 [15]. The proposed network outperformed SonoNet64 in classification by 0.4% in accuracy. The network parameter size of LPC-SonoNet was about one-third of the parameter size of SonoNet64. LPC-SonoNet had a lower inference time per sample than SonoNet64.

Figure 4 shows the confusion matrix of the classification on six categories by the proposed network and SonoNet64. It can be seen that, compared with SonoNet64, the proposed network performed better in the detection of the fetal abdomen and fetal thorax but worse in the detection of the fetal femur and other.

Table 6 compares the proposed network with state-of-the-art ensemble networks using Burgos-Artizzu et al.’s dataset [22] in terms of accuracy, optimizer, data augmentation, and network parameters. Due to the fact that the code and network parameters in the compared networks are not available, the parameter sizes of these ensemble networks were roughly estimated based on their network architectures. Although there was no specific evidence that the proposed network outperformed the compared networks in standard plane detection accuracy due to different experimental settings, it was demonstrated that the proposed network had a much smaller parameter size than the compared networks with an acceptable detection performance. In addition, the compared networks did not use data augmentation.

### 3.2. Results of Nine-Category Classification

**Figure 5:**
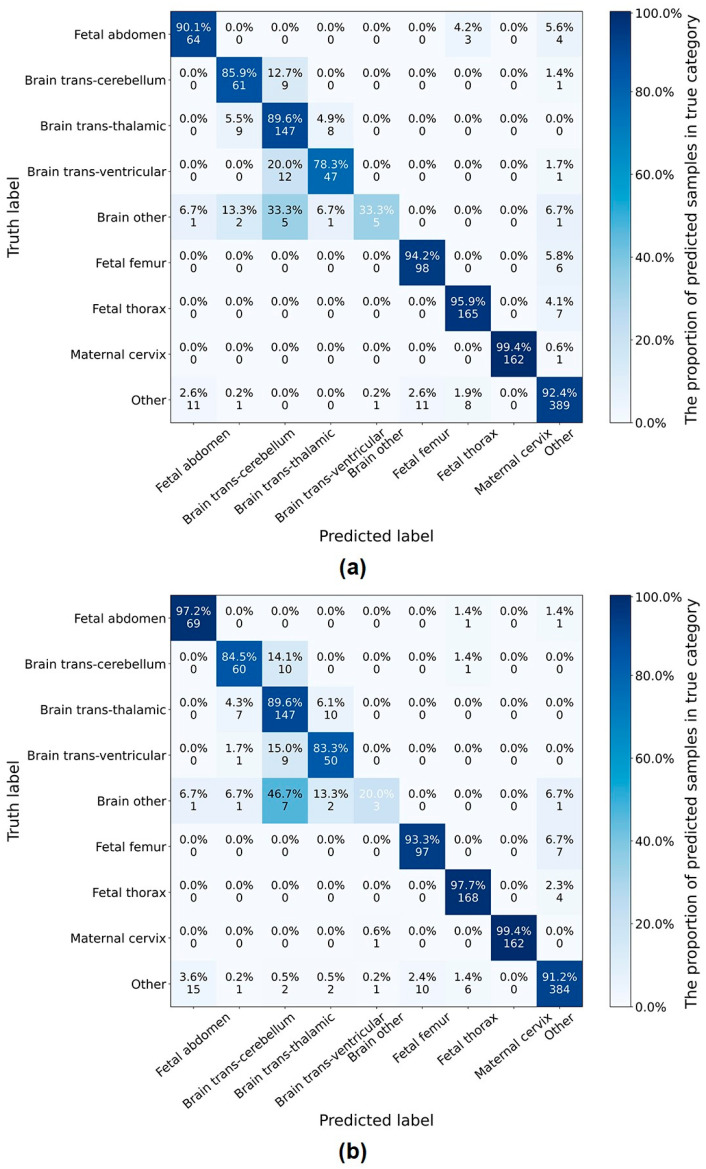
The confusion matrix of nine-category classification of standard planes by the proposed network (**b**) and SonoNet64 (**a**).

Table 7 shows the results of the classification on nine categories by the proposed LPC-SonoNet and SonoNet64 [15]. The proposed network outperformed SonoNet64 in classification by 0.2% in accuracy, but with a slightly lower sensitivity. Figure 5 shows the confusion matrix of the classification on nine categories by the proposed network and SonoNet64. In the classification of nine categories, the performance was improved in the detection of the fetal abdomen but reduced in the fetal thorax and other, compared with the classification of six categories. In the detection of four categories of fetal brain standard planes, the proposed network performed best in the detection of the trans-thalamic but worst in the detection of the other.

## 4. Discussion

In this paper, we incorporated the LPC blocks into SonoNet64 and the proposed LPC-SonoNet for fetal ultrasound standard plane detection. The proposed network replaced the convolutional blocks of SonoNet64 with the LPC blocks. The pyramid architecture of the LPC blocks could leverage features from various scales and fuse them with few convolutional layers. The proposed LPC-SonoNet model was trained and tested on a public dataset containing six categories of standard planes, i.e., Burgos-Artizzu et al.’s dataset [22]. Experimental results showed that LPC-SonoNet slightly outperformed SonoNet64 with much fewer network parameters. In addition, we further divided the dataset into nine categories and pioneered the nine-category classification using LPC-SonoNet, with a promising detection performance. This study has provided a lightweight network for deep-learning-based fetal ultrasound standard plane detection.

Compared with the convolutional layers in SonoNet64, the pyramid architecture in the proposed LPC-SonoNet enables most convolutional layers to process tensor data with less channels. The average number of channels of tensor data that SonoNet64 needs to process is about 307, while the counterparts of the proposed network is 230. Therefore, the proposed network has a much smaller parameter size than SonoNet64. However, the small parameter size may lead to disadvantages such as low sensitivity in the classification of nine categories (Table 7). In addition, the proposed network had less satisfying performance in the category of other brain standard planes possibly due to the small image size of this category.

In previous work, the ensemble networks tended to combine the predictions of various base networks such as VGG [30] and ResNet [33] and concluded the final prediction. For example, the three networks proposed by Krishna and Kokil [5,23,24] combined the feature vectors form VGG-19, ResNet-50, AlexNet, and DarkNet19 and fused these vectors with support vector machines or multi-layer perceptron. The network proposed by Sindhu et al. combined InceptionResNetV2, DenseNet121, and Xception [27]. The architecture of these ensemble frameworks did leverage the features from different scales, bringing excellent classification performance but leading to larger model parameter sizes and the requirement for powerful hardware [5]. In contrast, there was only one single base network in the proposed LPC-SonoNet and this design resulted in much smaller parameter sizes (Table 6).

In order to address the data imbalance issue in Burgos-Artizzu et al.’s dataset [22], data augmentation was applied in this work. Table 8 compares the performance of the proposed network trained with and without data augmentation in the classification of six categories of standard planes. It can be seen that the data augmentation slightly improved the performance in classification. Note that the compared methods in Table 6 have not used data augmentation. We argue that, if these methods have used data augmentation, their performance may be improved. In addition, the compared methods in Table 6 used stochastic gradient descent with momentum (SGDM) as the optimizer. In this study, we used the Adam optimizer because we experimentally found that it yielded better performance for the proposed LPC-SonoNet than SGDM.

As described in Section 1, previous studies have focused on classifying either six or three categories, without nine categories. This study pioneers the classification of nine categories. The possible reason why previous studies have not considered nine-category classification may lie in the fact that the sub-categories of the fetal brain standard plane, particularly for the fetal trans-ventricular standard plane and the other brain standard plane (Table 2), have relatively small sizes of images. This issue may pose challenges for the direct classification of nine categories. In this study, such an issue has been addressed by the data augmentation method (Table 4).

**Figure 6:**
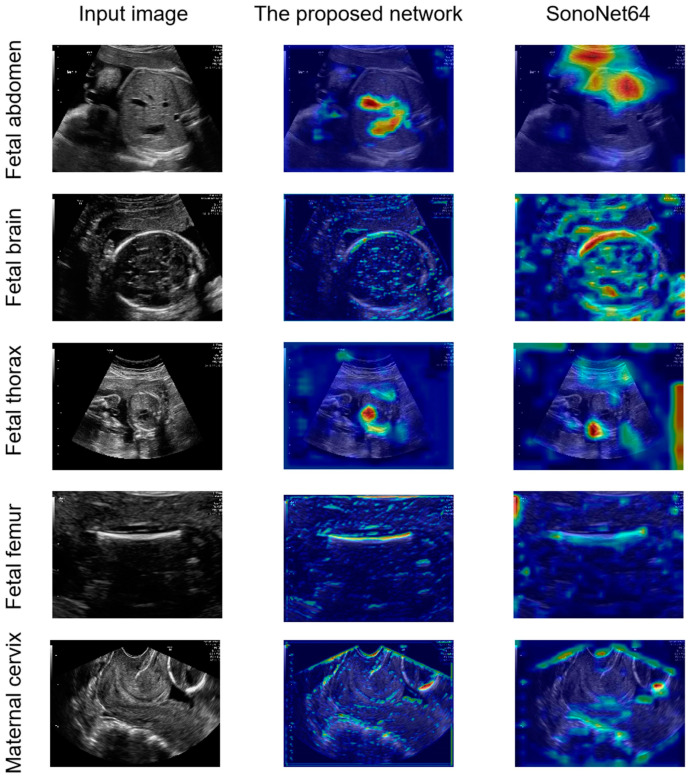
The comparison of heatmaps from the proposed network and SonoNet generated by the gradient-weighted class activation mapping (GradCAM) [34] technique.

Compared with SonoNet64, the proposed network had a better ability in the detection of the fetal abdomen and fetal thorax standard planes (Figure 4 and Figure 5). To explore the interpretability of the proposed LPC-SonoNet and SonoNet64, the gradient-weighted class activation mapping (GradCAM) [34] technique was used, and the heatmaps generated with GradCAM which used warm color to depict the attention of the network on the input data are shown in Figure 6. The heatmaps of LPC-SonoNet are more concentrated in the relevant regions of the fetal abdomen and fetal thorax standard planes than SonoNet64. It is possible that the pyramid architecture in LPC blocks enable the proposed network to have a large receptive field so that it can focus on the right regions related to the class of standard plane. However, this architecture makes the proposed network ignore the boundary of tissue so that the proposed network performed worse than SonoNet64 in the detection of the outlines of the skull and femur which is important in the classification of brain and femur standard planes.

This study has limitations. First, LPC-SonoNet has the limitation of a weak generalization ability. Trained on the high-quality images from Burgos-Artizzu et al.’s dataset which are collected with the devices such as Voluson S8 and Voluson S10 [22], the proposed LPC-SonoNet performed much worse on the low-quality images from another public dataset by Sendra-Balcells [35] (Table 9). This kind of reduction in detection accuracy can also be observed for SonoNet64 in Table 9. The reason may be that the Sendra-Balcells dataset [35] is quite different from Burgos-Artizzu et al.’s dataset [22]. The images in the Sendra-Balcells dataset [35] were collected with devices including but not limited to Mindray DC-N2 and Voluson P8 in resource-limited countries including Algeria, Egypt, and Malawi. The categories of images from this dataset included the fetal abdomen, fetal brain, fetal thorax, and fetal femur. Secondly, although the parameter size of the proposed network is much less than SonoNe64, it fails to significantly reduce the inference time (Table 5). It is probable that the frequent searching and merging for tensors in the concatenation function consumes much time for LPC-SonoNet. In future work, the generalization ability of the proposed LPC-SonoNet can be improved by methods such as adding low-quality images into the training set. In addition, the inference time may be further decreased, possibly by optimizing the architecture of the network and decreasing the number of concatenation layers.

## 5. Conclusions

In this paper, we introduced the LPC block into the classical SonoNet and proposed LPC-SonoNet with reduced network parameters for fetal ultrasound standard plane detection. A publicly available dataset containing six categories of ultrasound images was used. The dataset was further divided into nine categories. The proposed LPC-SonoNet model was trained on the augmented training set and tested on the test set. Data augmentation conducted on the training set addresses the data imbalance issue. The experimental results showed that the proposed LPC-SonoNet slightly outperformed SonoNet in standard plane detection accuracy, with a much smaller number of network parameters. In addition, we have pioneered the nine-category classification of standard planes, also with promising performance. The proposed LPC-SonoNet may be used as a lightweight network for fetal ultrasound standard plane detection.

## Figures and Tables

**Table 1:** The number of images in the training set, validation set, and test set for the six categories of fetal ultrasound standard planes in Burgos-Artizzu et al.’s dataset [22].

Six-Category Standard Planes	Total	Training Set	Validation Set	Test Set
Fetal abdominal standard plane	711	568	72	71
Fetal brain standard plane	3092	2472	310	310
Fetal femur standard plane	1040	832	104	104
Fetal thorax standard plane	1718	1374	172	172
Maternal cervix	1626	1300	163	163
Other	4213	3370	422	421

**Table 2:** The number of images in the training set, validation set, and test set for the nine categories of fetal ultrasound standard planes in Burgos-Artizzu et al.’s dataset [22].

Nine-Category Standard Planes	Total	Training Set	Validation Set	Test Set
Fetal abdominal standard plane	711	568	72	71
Fetal trans-ventricular standard plane	597	477	60	60
Fetal trans-thalamic standard plane	1638	1310	164	164
Fetal trans-cerebellum standard plane	714	571	72	71
Other brain standard plane	143	114	14	15
Fetal femur standard plane	1040	832	104	104
Fetal thorax standard plane	1718	1374	172	172
Maternal cervix	1626	1300	163	163
Other	4213	3370	422	421

**Table 3:** The number of images in the training set for the classification of six categories before and after data augmentation.

Six-Category Standard Planes	Before Augmentation	After Augmentation
Fetal abdominal standard plane	568	2840
Fetal brain standard plane	2472	2472
Fetal femur standard plane	832	2496
Fetal thorax standard plane	1374	2748
Maternal cervix	1300	2522
Other	3370	3370
Total	9916	16,448

**Table 4:** The number of images in the training set for the classification of nine categories before and after data augmentation.

Nine-Category Standard Planes	Before Augmentation	After Augmentation
Fetal abdominal standard plane	568	1300
Fetal trans-ventricular Standard plane	477	1300
Fetal trans-thalamic standard plane	1310	1310
Fetal trans-cerebellum standard plane	571	1300
Other brain standard plane	114	1368
Fetal femur standard plane	832	1300
Fetal thorax standard plane	1374	1374
Maternal cervix	1300	1300
Other	3370	3370
Total	9916	13,922

**Table 5:** The results of the proposed LPC-SonoNet and SonoNet64 [15] in the classification of six categories of standard planes.

Method	Accuracy	Sensitivity	Specificity	Parameters (million)	Inference Time per Sample (ms)
SonoNet64 [15]	96.6%	95.4%	99.2%	14.9	21.8
The proposed network	97.0%	96.4%	99.43%	4.3	20.0

**Table 6:** The comparison of the proposed network with state-of-the-art ensemble networks using Burgos-Artizzu et al.’s dataset [22] in terms of accuracy, optimizer, data augmentation, and network parameters. SGDM: stochastic gradient descent with momentum.

Method	Accuracy	Optimizer	Data Augmentation	Parameters (million)
Krishna and Kokil [23]	95.1%	Not reported	No	44.0
Krishna and Kokil [24]	95.5%	SGDM	No	78.8
Krishna and Kokil [5]	95.7%	SGDM	No	98.6
Annamalai and Sindhu [27]	96.9%	Not reported	No	12.9
The proposed network	97.0%	Adam	Yes	4.3

**Table 7:** The results of the proposed LPC-SonoNet and SonoNet64 [15] in the classification of nine categories of standard planes.

Method	Accuracy	Sensitivity	Specificity
SonoNet64 [15]	91.7%	84.4%	98.9%
The proposed network	91.9%	84.0%	99.0%

**Table 8:** The performance of the proposed network trained with and without data augmentation in the classification of six categories of standard plane.

Data Augmentation	Accuracy	Sensitivity	Specificity
No	96.4%	95.7%	99.3%
Yes	97.0%	96.4%	99.4%

**Table 9:** The accuracy of the proposed network and SonoNet64 trained on Burgos-Artizzu et al.’s dataset [22] in the classification of six categories on the low-quality dataset by Sendra-Balcells [35] consisting of three subsets.

Origin of the Subset	SonoNet64	The Proposed Network
Algeria	78.0%	69.0%
Egypt	63.0%	58.0%
Malawi	75.0%	74.0%

## Data Availability

The dataset used in this study is publicly available. The code of LPC-SonoNet will be made available publicly at https://github.com/ytx-pedestrian/LPC-SonoNet (accessed on 20 March 2024).
